# A Rare Presentation of Polypoid Endometriosis of the Douglas Pouch: Case Report

**DOI:** 10.5334/jbsr.3420

**Published:** 2024-02-13

**Authors:** Emilie Demondion, Yves Borghesi, Nathalie Trouillet

**Affiliations:** 1 Medical imaging department, Hôpital Jean Bernard, Valenciennes, France; 2 Gynaecological surgery department, Hôpital Jean Bernard, Valenciennes, France; 3 Anatomical Pathology department, Hôpital Jean Bernard, Valenciennes, France

**Keywords:** Endometriosis, magnetic resonance imaging (MRI), ovarian neoplasms, granulosa cell tumor, pelvis neoplasm

## Abstract

A case is reported of a 46-year-old woman referred to a magnetic resonance imaging (MRI) for menometrorrhagia. MRI revealed a mass lesion lateral to the uterus fundus, suspicious of an ovarian granulosa cell tumor. Extensive surgery was performed. Histological examination revealed a polypoid endometriosis lesion arising from the Douglas pouch.

*Teaching point:* Polypoid endometriosis is a rare benign entity with a challenging differential diagnosis from malignancy. Specific MRI features can contribute to the diagnosis and thus avoid excessive surgical resection.

## Introduction

Polypoid endometriosis is a rare variant of endometriosis first described in 1980 [1] and refers either to lesions that histologically look like endometrial polyps or to lesions that are histologically similar to endometriosis but with an exophytic growth [2].

These nodular lesions may occur at either surface of the ovary, digestive tract, uterine serosa, bladder, ureter, or pelvis peritoneum.

We report a case of a polypoid endometriosis nodule arising from the Douglas pouch incidentally discovered on magnetic resonance imaging (MRI).

## Case report

A 46-year-old female was referred for an MRI for menometrorrhagia. Her medical past history and pelvic examination were unremarkable. She had been using oral contraceptives for years.

**Figure 1:**
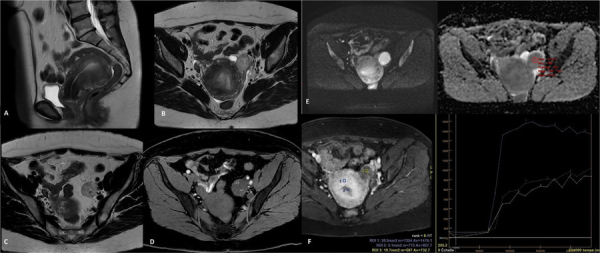
MRI appearance. (A) Sagittal T2 image showing thickening of the junctional zone. (B–D) Axial T2 weighted images and axial T1 weighted fat suppressed images showing a left latero uterine mass containing spots of hemorrhagic content. (E) Diffusion-weighted images (*b* = 1,000) showing no diffusion restriction. ADC-mean value of 1.4×10^−3^ mm^2^/s. (F) Enhancement curve of the lesion compared to that of myometrium and endometrium.

MRI (1.5T) showed a solid lesion left to the uterus, measuring 40 mm, with homogeneous intermediate T2-signal intensity similar to endometrium and hyperintense T1 spots after fat suppression, consistent with hemorrhagic content (Figure 1). This lesion seemed to be distinct from the left ovary and, no pedicle attached it to the uterus.

No peritoneal carcinosis nodule and no lymphadenopathy were associated. The lesion showed no diffusion restriction (ADC 1.4) and progressive enhancement (type 1 curve compared with that of uterine myometrium), similar to that of endometrium (Figure 1). It was associated with thickening of the junctional zone, consistent with diffuse adenomyosis.

In this context of adenomyosis associated with a solid homogeneous mass that presented hemorrhagic changes, the lesion was suspected to be an ovarian Granulosa cell tumor.

**Figure 2:**
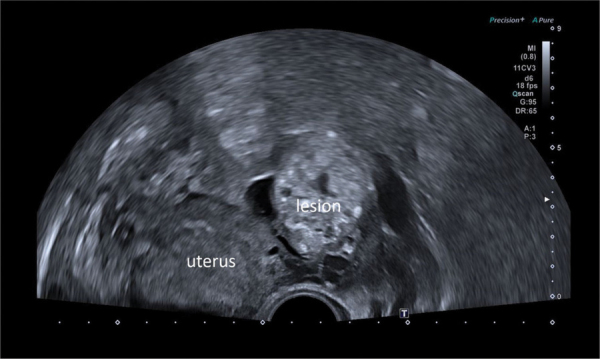
Ultrasound showing nodular lesion with hyperechoic spots and cyst-like areas. Lateral to the uterus.

Transvaginal ultrasound performed after MRI demonstrated that the lesion was mobilized when moving the ovary. The mass was diffusely hyperechoic, with the presence of a few bright spots that could correspond to microcysts (Figure 2).

**Figure 3:**
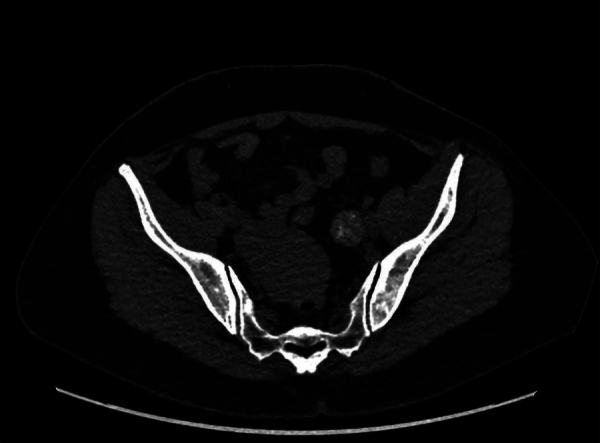
Unenhanced pelvic CT-scan illustrating the nodule containing hyperdense spots.

The staging computed tomography (CT) scan was negative and showed spontaneously dense spots in the mass on unenhanced scans, consistent with hemorrhagic changes (Figure 3).

**Figure 4:**
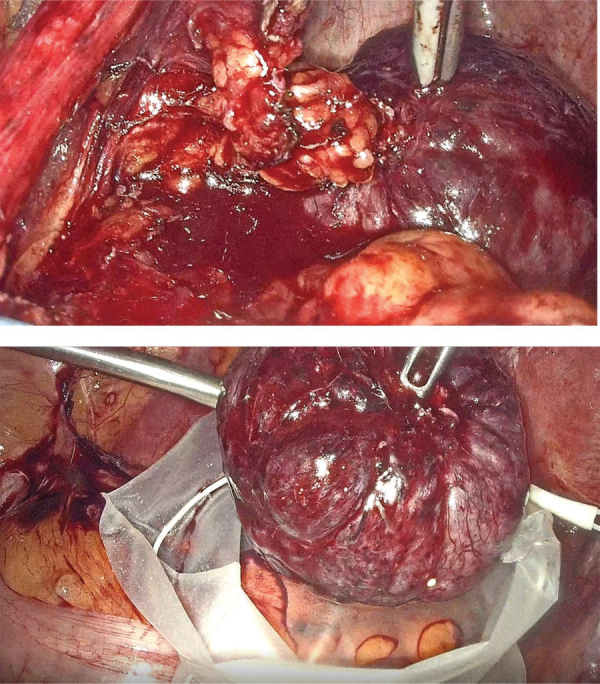
Photographs of the resected mass from the Douglas pouch.

After clinical case discussion, the patient underwent extensive surgery (hysterectomy and left salpingo-oophorectomy) on the hypothesis of a malignant ovary tumor. A nodular lesion arising from the Douglas pouch was found, bleeding upon contact. There was no lesion arising from the ovary, fallopian tube, or uterus (Figure 4).

**Figure 5:**
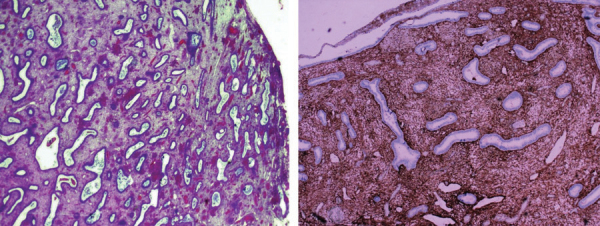
Photomicrographs of the resected mass (hematoxylin and eosin stain on the left and immunochemistry CD 10 marker on the right) showing endometrial glands and endometrial stroma.

Histopathological examination revealed a benign polypoid endometriosis nodule characterized by dilated endometrial glands with some cystic areas and a stromal component positive to CD10 in immunohistochemistry demonstrating its endometrial origin (Figure 5).

## Discussion

Polypoid endometriosis is a benign and distinctive form of pelvic endometriosis not well known as it is rather uncommon. Only a limited cohorts are reported in the literature studying its histopathological and clinical aspects [2].

The differential diagnosis of malignancy is raised [3,4] as it can mimic ovarian tumors with peritoneal dissemination imaging and surgery.

MRI features are not fully defined, rendering preoperative diagnosis difficult, especially in the absence of a known history of endometriosis.

MRI characteristics found in the literature include polypoid lesions either single or multiple, often appearing hyperintense on T2-weighted sequences due to cystic glands dilatation [4-6].

Another MRI feature frequently reported is the presence of foci of hyperintensity on T1-fat suppressed sequences reflecting hemorrhage [7] and a peripheral T2 black rim sign demonstrating fibrous tissue surrounding the lesion [8].

No additional signs of malignancy are seen: no lymphadenopathy, high apparent diffusion coefficient (ADC) values on diffusion weighted imaging (DWI) and moderate enhancement on dynamic contrast enhanced-MRI (DCE-MRI).

Coexisting adenomyosis is found to be frequent [8]. Interestingly, polypoid endometriosis commonly affects peri- to postmenopausal women, while traditional endometriosis predominates in premenopausal women.

The present case is unique with regards to the completeness of its multimodality exploration.

In conclusion, the definition of MRI-specific characteristics could help to evoke the benignity of the lesion and prevent overtreatment, especially in women of childbearing age.
